# Genome-wide characterization of Plasmodium vivax infections in local travelers and non-travelers from the Peruvian Amazon

**DOI:** 10.21203/rs.3.rs-10164856/v1

**Published:** 2026-07-07

**Authors:** Mahdi Safarpour, Luis Cabrera-Sosa, Johanna H. Kattenberg, Ramses Deyaert, Anna Rosanas-Urgell, Joseph M. Vinetz, Jean-Pierre Van geertruyden, Dionicia Gamboa, Christopher Delgado-Ratto

**Affiliations:** University of Antwerp; University of Antwerp; Instituut voor Tropische Geneeskunde; Instituut voor Tropische Geneeskunde; Instituut voor Tropische Geneeskunde; Yale University; University of Antwerp; Universidad Peruana Cayetano Heredia; University of Antwerp

**Keywords:** Genomic surveillance, imported malaria, molecular epidemiology, population genomics, genetic markers

## Abstract

In areas progressing toward malaria elimination, distinguishing locally acquired from imported malaria infections is essential for targeted surveillance. We used whole-genome sequencing (WGS) to characterize *Plasmodium vivax* infections in individuals with reported local travel and non-travelers in three communities in the Peruvian Amazon: Libertad (n = 24), Gamitanacocha (n = 23), and Urco Miraño (n = 9). Among 56 confirmed *P. vivax* infections, 16 were identified in local travelers. DNA was extracted from whole blood, selectively amplified, and parasite species were confirmed by qPCR. Genetic diversity was assessed using expected heterozygosity and nucleotide diversity, genetic differentiation using Fst, population structure using PCA, DAPC, and ADMIXTURE, and relatedness using IBD analysis. Traveler-associated infections in Libertad showed higher diversity than non-travelers (mean He = 0.432 vs 0.304; mean π = 0.290 vs 0.168). Pairwise Fst showed low differentiation between Libertad and Gamitanacocha (Fst = 0.056), but higher differentiation between Urco Miraño and Libertad (Fst = 0.548) and Gamitanacocha (Fst = 0.773). PCA and DAPC showed clear clustering of Urco Miraño parasites, while Libertad and Gamitanacocha partially overlapped. Traveler-associated infections did not form distinct clusters and mostly remained connected to non-traveler infections. ADMIXTURE indicated greater heterogeneity among travelers, and IBD identified two unconnected traveler-associated infections overall. These findings support integrating genomic and epidemiological data to interpret mobility-associated malaria transmission.

## Introduction

Human mobility is widely recognized as a key determinant of malaria transmission dynamics and parasite population structure. The movement of infected individuals from source areas can introduce new parasite lineages into sink areas, sustain residual transmission, and undermine local elimination efforts [1, 2]. Genetic studies have further shown that human mobility shapes parasite gene flow, influences spatial genetic structure, and contributes to the spread of drug-resistant variants [3, 4]. In low-transmission settings, where local transmission chains are fragmented, even a small number of imported infections may re-establish outbreaks or maintain endemicity. As countries transition from control to elimination, accurately identifying imported versus locally acquired infections has become a central priority for surveillance systems [5].

Over the past two decades, different molecular tools have been developed to characterize parasite diversity and connectivity. Microsatellite markers were initially used to assess genetic diversity and infer transmission intensity [6, 7]. Subsequently, standardized single-nucleotide polymorphism (SNP) barcodes provided higher resolution for studying parasite population genetics at both regional and global scales. They also enabled investigation of drug resistance and facilitated analyses that are difficult to perform using microsatellite markers alone, such as identity-by-descent (IBD) analysis, which requires a large number of genetic variants across the genome [8, 9]. These approaches have generated valuable insights into transmission patterns and have been incorporated into molecular surveillance frameworks. However, their resolution may be limited in settings with low parasite genetic diversity, potentially reducing their discriminatory power for identifying infection origin at fine spatial scales, such as between nearby communities or local transmission areas [5].

The advent of whole-genome sequencing (WGS) has transformed malaria population genomics by enabling genome-wide characterization of genetic variation, recombination, and relatedness. In this context, WGS provides high-throughput data that support both allele frequency-based analyses, such as heterozygosity and Fst, and haplotype-based approaches such as identity-by-descent (IBD), which are particularly informative for detecting recent shared ancestry [5, 9]. Using genome-wide data, studies have successfully reconstructed transmission networks, identified parasite movement, and characterized regional population structure in Africa and Southeast Asia [10–13]. These findings demonstrate the potential of WGS for tracking parasite flow across broad geographic regions.

Nevertheless, most successful applications of WGS for distinguishing parasite origins have been conducted across large geographic scales, such as among continents or countries, where parasite populations are genetically distinct. At finer spatial scales, particularly in areas with historically bottlenecked populations and partially clonal transmission, discrimination becomes more challenging. In South America, including the Peruvian Amazon, *P. vivax* populations have been shown to exhibit relatively low diversity and extensive relatedness due to demographic history and limited recombination [14, 15]. In such contexts, high genome-wide similarity between parasites from neighboring communities may complicate the interpretation of genetic clustering and reduce the ability to detect recent signals of importation.

In addition to the genetic similarity between parasite populations, the interpretation of genomic patterns also depends on the accuracy and completeness of epidemiological data. Incomplete travel reporting, recall bias, unreported mobility, or indirect travel routes may lead to misclassification of infections and further complicate the validation of genomic inferences regarding parasite origin [2]. This is particularly relevant in river-connected settings such as the Peruvian Amazon, where frequent and informal human mobility may not be fully captured by standard surveillance systems. The biological characteristics of *P. vivax* may also contribute to this uncertainty, as dormant liver-stage hypnozoites can reactivate weeks to months after the initial infection [16, 17]. Therefore, an infection detected after recent travel may reflect either a newly acquired infection during travel or the relapse of an earlier infection acquired locally [18].

In this context, the Peruvian Amazon presents an ideal setting to investigate the challenges of distinguishing traveler-associated from local malaria infections in parasite populations characterized by high relatedness, low diversity, and incomplete mobility data. River networks in the Peruvian Amazon facilitate frequent movement of people between communities, potentially promoting parasite gene flow. At the same time, the geographic isolation of some riverine communities may still generate localized parasite populations and fine-scale genetic structure. In this setting, WGS provides high-resolution genomic data that may help detect both parasite connectivity and hidden population differentiation, making it a valuable approach for investigating whether traveler-associated infections can be distinguished from local transmission.

In this study, we aimed to characterize and compare the genetic diversity and population structure of *P. vivax* infections in individuals with reported local travel and non-travelers using WGS data. By assessing the performance of WGS at a fine spatial scale characterized by high parasite relatedness and substantial genetic similarity, this study provides insight into both the potential and the limitations of genomic approaches for distinguishing traveler-associated from locally acquired infections. The findings of this study may contribute to the development of genomic surveillance approaches for tracking parasite gene flow, identifying transmission hotspots, and improving targeted malaria elimination strategies in low-transmission settings.

## Methods

### Study Setting and Design

**Figure 1:**
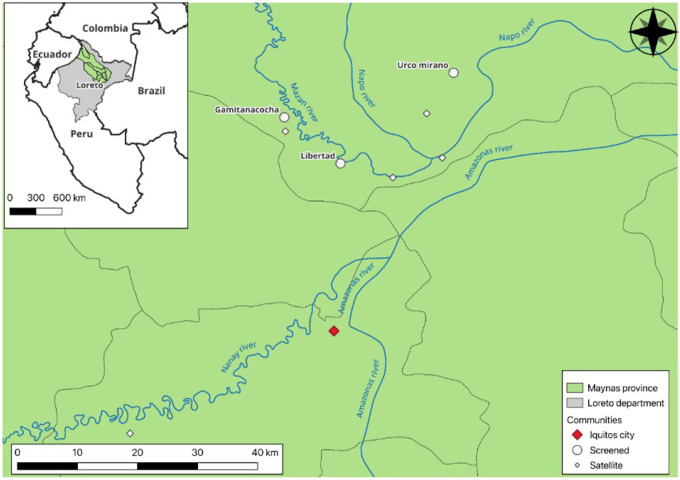
Study area and sampling locations in the Peruvian Amazon. Circles indicate the communities where samples were collected, while diamond symbols represent reported travel destinations of individuals with travel history. Iquitos city is shown by a red diamond. The map was created using QGIS version 3.40 (QGIS Development Team, 2025; QGIS Geographic Information System, Open Source Geospatial Foundation Project; http://www.qgis.org/). Administrative boundaries for Peru were obtained from open-access sources, including the National Institute of Statistics and Informatics (INEI), the National Geographic Institute of Peru (IGN), and the Spatial Data Infrastructure of Peru (IDEP). Country-level boundaries for neighboring countries were obtained from the open-access Natural Earth database. Hydrographic layers were obtained from IDEP and IGN.

This study analyzed human blood samples collected by the ICEMR 2.0 P1 between 2021 and 2022 in three malaria-endemic communities of the Mazan district, Loreto region in the Peruvian Amazon: Libertad, Gamitanacocha, and Urco Miraño (Fig. 1). Libertad is a riverine community characterized by its proximity to river systems that provide suitable habitats for malaria vectors. The community also has a highly mobile population, making it an important setting for malaria surveillance and control efforts.

Gamitanacocha is located upstream along the same river system (Mazan river) that connects it to Libertad and experiences elevated malaria transmission levels. Previous studies have highlighted the important role of human mobility in shaping malaria transmission in this region, with frequent travel between riverine communities and Mazán town potentially facilitating parasite gene flow between communities [19]. Urco Miraño is relatively more isolated compared with Libertad and Gamitanacocha. Unlike these two communities, Urco Miraño is located along the Napo River and has only indirect river access to the other communities. Like Libertad and Gamitanacocha, it faces persistent challenges related to malaria transmission due to its ecological setting and limited accessibility. Studies in the Peruvian Amazon, particularly in the Mazán River basin, have shown that riverine and peri-river communities such as Urco Miraño, characterized by proximity to slow-moving water bodies and forest fringe environments, tend to harbor higher densities of *Anopheles darlingi*, the primary malaria vector. Similar patterns have been reported in communities around Iquitos and along the Nanay and Napo river systems, where ecological conditions favor vector breeding and sustain higher malaria transmission [20].

During the ICEMR project, population surveys in these communities were designed to characterize parasites at individual and population levels [21]. Among 7,915 surveyed individuals, 56 confirmed malaria infections were included in this study. Individuals were classified as travelers if they reported staying outside their community for at least one night during the past month. Overall, 16 individuals had recent travel history (travelers), while the remaining (n = 40) had no reported travel (non-travelers).

### Ethics statement

The present study was approved by the Ethics Committee of Antwerp University Hospital (UZA)/University of Antwerp (Project ID 3343). The FWO project was also approved by the Ethics Committee of Universidad Peruana Cayetano Heredia (UPCH; SIDISI code 208631). This study used samples collected as part of the ICEMR 2.0 P1 project, “Transmission Dynamics of Residual and Re-emerging Malaria in Amazonia: Defining Pathways Towards Malaria Elimination.” The study protocol, informed consent forms, and assent forms were approved by UPCH (SIDISI code 101518). Written informed consent was obtained from all participants or their legal guardians, with assent obtained from minors when applicable. Laboratory work and sample processing at the Institute of Tropical Medicine (ITM)-Antwerp were approved under reference number 1818/24. All procedures were conducted in accordance with relevant institutional guidelines and Peruvian Ministry of Health regulations.

### Sample collection and Microscopy

Whole blood samples were collected from participants aged ≥ 3 months. Malaria cases were identified through both active and passive case detection. For passive case detection, microscopy diagnosis using thick and thin blood smears was performed at local health centers, whereas for active case detection, blood smears were examined by the field team in each community [21]. All microscopy procedures followed the Peruvian Ministry of Health (MINSA) guidelines, which are based on WHO protocols [22]. All blood samples were subsequently transported to the Universidad Peruana Cayetano Heredia (UPCH), Lima, Peru, where molecular diagnosis was performed on all samples using real-time PCR, regardless of microscopy result. Samples confirmed as *Plasmodium*-positive by PCR were selected for further analysis. From these, 200 μL of whole blood was shipped to ITM-Antwerp.

### DNA Extraction

DNA was extracted from 200 μL of whole blood at the Institute of Tropical Medicine (ITM), Antwerp, using the QIAamp DNA Blood Kit (Qiagen, Hilden, Germany), following the manufacturer’s protocol. The extracted DNA was eluted in 50 μL of Tris-EDTA (TE) buffer and stored at − 20°C until further processing.

### Molecular Diagnosis

All samples were initially validated at UPCH using qPCR with high-resolution melting assays and TaqMan probes [23]. To ensure sample integrity, consistency after storage and shipment, and to confirm the mono-species infections, qPCR was repeated at ITM following DNA extraction as part of the standard workflow. *P. vivax* infections were confirmed by targeting the mitochondrial cox1 gene (mtCOX1) [24]. Samples with undetectable Cycle Threshold (Ct) values were excluded. No predefined Ct threshold was applied; therefore, samples with high Ct values (> 35), were kept maximizing sample inclusion given the low parasite densities observed. Absolute quantification of parasite DNA concentration (parasites/μL) was performed using Qubit dsDNA High Sensitivity (HS) Assay Kit (Thermo Fisher Scientific, Waltham, MA, USA) on a Qubit Fluorometer.

### Selective Whole Genome Amplification (sWGA)

Parasite DNA was selectively amplified using sWGA primers targeting conserved regions of the Plasmodium genome, originally described by Cowell et al. [25], using a modified protocol applied at ITM-Antwerp. Two sets of primers were used to maximize genome coverage. The amplified DNA was purified with AMPureXP beads (Beckman Coulter, Brea, California, USA).

### Sample selection and sequencing workflow

A total of 166 blood samples were initially included for laboratory processing, consisting of 86 samples from Libertad, 60 from Gamitanacocha, and 20 from Urco Miraño. Among these, 41 samples were from individuals with reported travel history. After selective whole-genome amplification, 10 samples were excluded because they did not meet the minimum DNA quantity requirement of 200 ng in 20–25 μL, leaving 156 samples for pre-library quality control (Supplementary Figure S1).

### Whole genome sequencing

The sWGA products meeting the minimum input requirement for Watchmaker PCR-free library preparation, defined by Macrogen Europe as ≥ 200 ng DNA in 20–25 μL were sent to Macrogen Europe (Amsterdam, Netherlands) for low-input library preparation and sequencing. During pre-library DNA quality control, DNA quantity and DNA Integrity Number (DIN) were assessed using TapeStation (Agilent Technologies, Santa Clara, CA, USA). At this step, 49 samples failed due to DNA quantity below 0.2 μg and/or DIN below 7. The remaining 107 samples proceeded to library preparation using the Watchmaker PCR-Free protocol (Illumina, San Diego, CA, USA), followed by fragment size quality control using TapeStation. Samples with library concentrations below 5 nM were considered low quantity and excluded from sequencing. Following this step, 12 additional samples were excluded, resulting in 95 samples that were sequenced using NovaSeq platform (Illumina, San Diego, CA, USA), generating 150 bp paired-end reads with an average coverage of ~ 100× per sample. After post-sequencing quality control, 89 samples achieved Q30 > 90% (Supplementary Figure S1).

## Data analysis

### Sequence data processing and variant calling

Raw paired-end sequencing reads were assessed for quality using FastQC. Adapter sequences and low-quality bases were trimmed using fastp, followed by a second round of FastQC to confirm improvement in read quality (Supplementary Figure S2). For alignment, reads were first mapped to the human reference genome (GRCh38) to remove host-derived sequences. Unmapped reads were subsequently aligned to the *P. vivax* reference genome (PvPAM, PlasmoDB release 68) [26] using BWA-MEM. Resulting alignments were sorted and PCR duplicates were identified and marked. Variant calling was performed using the Genome Analysis Toolkit (GATK). For each sample, variants were called per genomic region using HaplotypeCaller in GVCF mode, generating intermediate GVCF files. These were combined across samples using GenomicsDBImport, followed by joint genotyping with GenotypeGVCFs to produce cohort-level variant call sets.

To improve variant quality, SNPs and indels were processed separately. Hard filtering was applied to SNPs using the following criteria: Quality by Depth (QD) < 5.0, variant quality (QUAL) < 30.0, Strand Odds Ratio (SOR) > 3.0, Fisher Strand bias (FS) > 60.0, Mapping Quality (MQ) < 40.0, MQRankSum < − 12.5, and ReadPosRankSum < − 8.0. Variants failing these thresholds were excluded. Filtered SNPs and indels were then combined, and regional new variant call files were merged to generate a final genome-wide VCF file. Functional annotation of variants was performed using *snpEff* (PlasmoDB v68 database). Annotated VCF files were subsequently converted into tabular format for downstream analyses.

### Overall variant counts and polymorphic biallelic SNPs

The initial VCF file contained 400,536 variant records, including 240,653 biallelic SNPs, 9,861 multiallelic SNPs, and 150,022 indels. After applying variant-level quality filters, the filtered VCF file retained 334,365 variant records. These included 206,581 biallelic SNPs, 7,634 multiallelic SNPs, and 120,150 indels. For downstream population genetic analyses, only biallelic SNPs were retained. Samples with more than 40% missing genotypes were excluded before further SNP filtering, leaving 56 samples for downstream analysis. After removing SNPs with missingness greater than 50%, 19,297 biallelic SNPs remained. Among these, 16,485 SNPs were polymorphic, while 2,812 SNPs were monomorphic.

### Analytical framework for comparing traveler-associated and non-traveler infections

The population genetic analyses were used to assess whether traveler-associated infections showed genomic patterns distinct from non-traveler infections. Specifically, we evaluated whether traveler-associated infections: (i) formed separate genetic clusters in PCA and DAPC analyses, (ii) showed distinct ancestry profiles in ADMIXTURE analysis, and (iii) displayed relatedness to local parasite populations in IBD analysis. These results were compared and interpreted together with reported travel history to assess whether WGS data could support the identification of mobility-associated *P. vivax* infections.

### Genetic diversity and differentiation

Genetic diversity was measured as expected heterozygosity (He) using only 2632 polymorphic biallelic SNPs after excluding rare variants with global minor allele frequency (MAF) < 0.05. The calculation was done using the formula: He = [n/(n − 1)][1 − Σp2], where n is the number of genotyped samples and p is the frequency of each allele at a given locus [27, 28].

Genetic differentiation was expressed as pairwise Fst values and 95% confidence interval (95% CI), calculated based on Weir and Cockerham’s method using the diveRsity package in R [29, 30].

### Nucleotide diversity (π)

Nucleotide diversity (π) was calculated using a filtered set of 16,485 high-quality biallelic SNPs. Estimates were obtained using VCFtools from the VCF file, and π was computed in 500-bp sliding windows across the genome. The analysis was conducted for two datasets: (i) all samples within each community and (ii) the subset of infections reported in travelers. Distributions of per-site π values were visualized using violin plots to compare patterns of genetic variation across communities.

### Principal Component Analysis and Discriminant Analysis of Principal Components (DAPC)

PCA and DAPC were performed to explore genetic clustering patterns among malaria parasites. Genotype data from WGS were filtered to retain high-quality single-nucleotide polymorphisms (SNPs) with minor allele frequency ≥ 0.05 and missingness < 50%. PCA was conducted using SNPRelate R package (v1.42.1), with the first two principal components (PCs) used to visualize the separation of samples by community and travel history. DAPC was performed using the adegenet R package (v2.1.11). The optimal number of principal components retained in the analysis was selected using α-score optimization to minimize overfitting while maintaining discrimination between groups. Discriminant analysis was performed using community of origin as the grouping variable. The first two linear discriminants (LD1 and LD2) were then plotted to visualize sample clustering. In addition, loading plots for LD1 and LD2 were examined to assess the contribution of individual SNPs to population discrimination.

### Identity-by-Descent (IBD) Analysis

Genotype data were first converted into PED and MAP formats using VCFtools for IBD analysis. IBD sharing between pairs of *P. vivax* isolates was estimated using the isoRelate package. Genetic distances were calculated by converting physical positions to centimorgans (cM) using a recombination rate of 13.7 kb/cM [31]. Prior to analysis, genotype data were filtered to retain variants with a minor allele frequency ≥ 0.01, maximum missingness of 30% per isolate, and 60% per SNP. IBD segments were defined using a minimum of 450 SNPs and a minimum physical length of 700,000 bp.

### Admixture Analysis

Population structure and ancestry proportions were inferred using ADMIXTURE v1.3.0 [32]. Prior to analysis, genotype data were filtered for quality and analyzed using three datasets: (i) an unpruned dataset, (ii) a linkage disequilibrium (LD)-pruned dataset with r^2^ = 0.1, and (iii) an LD-pruned dataset with r^2^ = 0.2, generated using PLINK v1.9. The unpruned dataset was used to capture the full genome-wide signal, while LD-pruned datasets were used to reduce the influence of closely linked markers that could bias ancestry inference. Two LD thresholds were applied to assess the robustness of the inferred population structure under more stringent (r^2^ = 0.1) and more relaxed (r^2^ = 0.2) pruning conditions. This allowed us to determine whether the observed ancestry patterns were consistent across different levels of marker independence. Cross-validation (CV) was performed to determine the optimal number of ancestral populations (K), testing values from K = 2 to K = 10. For each K, ADMIXTURE was run with 100 bootstrap replicates and 5 independent iterations to ensure convergence and consistency of the ancestry estimates. The results were visualized in R (v4.4.2) using ggplot2 to generate ancestry proportion plots.

### Minimum spanning tree (MST) analysis

To explore the genetic relationships among *P. vivax* infections, pairwise genetic distances between samples were calculated using the metric 1 − PS, where PS represents the proportion of shared SNP alleles between two samples. A complete distance matrix was constructed from the SNP data, accounting only for loci with non-missing values in both samples. Missing pairwise comparisons were handled by substituting the maximum observed distance to maintain connectivity. Based on this distance matrix, a minimum spanning tree was generated, which connects all samples while minimizing the total genetic distance across the network. For visualization, nodes were colored according to community of origin, and travel status was indicated by overlaying a cross within the node for traveler-associated infections.

### Hierarchical clustering

For each pair of samples, the proportion of shared alleles (PS) was computed across all comparable SNP loci, excluding missing values. Genetic distance was then defined as 1 − PS, such that lower values indicate higher genetic similarity. A distance matrix was constructed from these pairwise comparisons and used for hierarchical clustering. Clustering was performed using the average linkage method (UPGMA) implemented in R (hclust). The number of clusters (K) was determined by inspecting the dendrogram structure and identifying stable groupings at different cut heights. Samples were annotated by community of origin using color coding, and travel history was indicated by marking traveler-associated infections with cross symbols.

## Statistical analysis

Expected heterozygosity (He) was compared across communities using the Kruskal–Wallis rank-sum test, followed by Dunn’s post hoc test for pairwise comparisons. For nucleotide diversity (π), the Friedman test was used because π values were compared across the same genomic windows among communities, treating each genomic window as a matched unit. Within each community, differences between traveler-associated and non-traveler infections were assessed using the Wilcoxon rank-sum test. P-values < 0.05 were considered significant. For multiple pairwise comparisons, P-values were adjusted using the Bonferroni correction.

## Results

### Demographic characteristics and local human mobility patterns

The study population included 402 participants from Urco Miraño, 385 from Libertad, and 107 from Gamitanacocha. Local travelers represented 633 of 894 participants (70.8%), while 261 participants (29.2%) were classified as non-travelers. The proportion of local travelers differed across communities (χ^2^ = 15.27, p < 0.001), with the highest proportion in Gamitanacocha (87/107, 81.3%), followed by Libertad (286/385, 74.3%) and Urco Miraño (260/402, 64.7%).

The cumulative proportion of PCR-confirmed *P. vivax* infection was higher among local travelers than non-travelers overall (99/633, 15.6% vs. 17/261, 6.5%; Fisher’s exact p < 0.001). In Libertad, local travelers had a higher cumulative proportion of infection than non-travelers (58/286, 20.3% vs. 4/99, 4.0%; p < 0.001). In Gamitanacocha, the proportions were similar between local travelers and non-travelers (32/87, 36.8% vs. 7/20, 35.0%). In Urco Miraño, the corresponding proportions were 9/260 (3.5%) among local travelers and 6/142 (4.2%) among non-travelers (Fisher’s exact p = 0.785).

The age distribution was similar across communities (mean range: 25.6–27.5). The sex distribution was also broadly balanced, although the proportion of females was higher in Gamitanacocha (56.1%) than in Libertad (45.7%) and Urco Miraño (46.3%). Overall, local travelers were older than non-travelers, with mean ages of 29.0 and 23.0 years, respectively. The sex distribution was similar between local travelers and non-travelers overall, with males representing 52.6% and 53.3% of each group, respectively. Within communities, local travelers were older than non-travelers in Gamitanacocha (26.3 vs. 22.6 years), Libertad (30.0 vs. 20.3 years), and Urco Miraño (28.9 vs. 24.9 years). In Gamitanacocha, females represented 52.9% of local travelers and 70.0% of non-travelers. In Libertad, males represented 55.2% of local travelers and 51.5% of non-travelers. In Urco Miraño, males represented 51.5% of local travelers and 57.7% of non-travelers.

Mazan was the most frequently reported travel destination in all three communities (Supplementary Figure S3). It accounted for 163 of 203 recorded destinations from Gamitanacocha (80.3%), 661 of 969 from Libertad (68.2%), and 465 of 695 from Urco Miraño (66.9%). Iquitos was the second most common destination shared across communities, accounting for 18 of 203 destinations from Gamitanacocha (8.9%), 78 of 969 from Libertad (8.0%), and 92 of 695 from Urco Miraño (13.2%).

Inter-community travel was less frequent. Travel from Gamitanacocha to Libertad was reported 7 times among 203 recorded destinations (3.4%), while travel from Libertad to Gamitanacocha was reported 3 times among 969 recorded destinations (0.3%). Travel from Libertad to Urco Miraño was reported once among 969 recorded destinations (0.1%), whereas travel from Urco Miraño to Libertad was not recorded. No travel was recorded between Gamitanacocha and Urco Miraño in either direction.

### Genetic diversity

**Figure 2:**
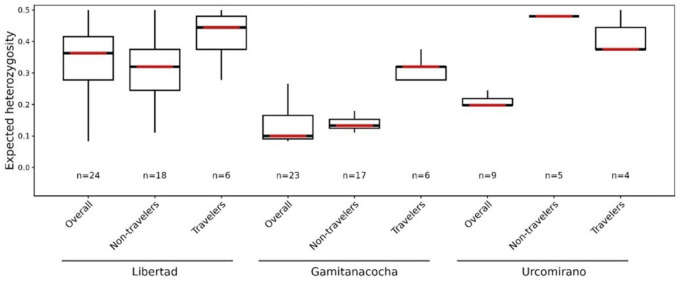
Genetic diversity patterns in Gamitanacocha, Libertad, and Urco Miraño. Expected heterozygosity (He) is shown for the overall population in each community, local infections only, and traveler-associated infections. Boxes represent the interquartile range with median values indicated by red lines; whiskers denote the range.

As shown in Fig. 2, Libertad showed the highest overall genetic diversity (median He = 0.363), followed by Urco Miraño (median He = 0.198), while Gamitanacocha showed the lowest diversity (median He = 0.1). Pairwise comparisons confirmed significant differences among all communities (p < 0.001). A similar pattern was observed among traveler-associated infections, with median He highest in Libertad (0.444), followed by Urco Miraño (0.375) and Gamitanacocha (0.320). Pairwise comparisons between traveler groups were significant for all community pairs (p < 0.001).

Within-community comparisons showed significant differences in He between travelers and non-travelers in all three communities. In Libertad, travelers had higher diversity (median He = 0.44) than non-travelers (median He = 0.32; p < 0.001) A similar pattern was observed in Gamitanacocha, where travelers showed higher diversity (median He = 0.320) than non-travelers (median He = 0.133; p < 0.001). In Urco Miraño, non-travelers showed higher diversity (median He = 0.480) than travelers (median He = 0.375; p < 0.001). However, this comparison should be interpreted carefully due to the very small group sizes (n = 5 non-travelers vs. n = 4 travelers).

### Nucleotide diversity (π)

**Figure 3:**
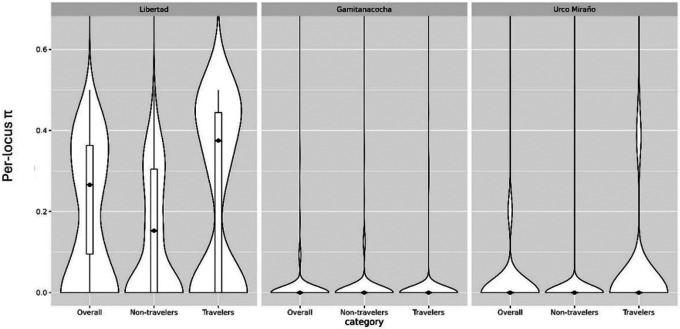
Per-locus nucleotide diversity (π) by community and travel status. Within each community, distributions are shown for the overall infected population, non-travelers, and traveler-associated infections.

In the overall population, π differed significantly between the three communities (Friedman test, χ^2^ = 3049.49, p < 0.001). Libertad showed the highest diversity (median π = 0.266), while diversity was much lower in Urco Miraño (median π = 0) and Gamitanacocha (median π = 0). All pairwise community comparisons were significant after multiple-testing correction (Fig. 3).

Among traveler-associated infections, nucleotide diversity also differed significantly between communities (Friedman test, χ^2^ = 2648.69, p < 0.001). Travelers from Libertad showed the highest diversity (median π = 0.375), followed by travelers from Urco Miraño (median π = 0), while travelers from Gamitanacocha showed the lowest diversity (median π = 0). All pairwise comparisons between traveler groups were significant.

Within communities, the relationship between travel status and nucleotide diversity varied. In Libertad, travelers had higher diversity (median π = 0.375) than non-travelers (median π = 0.153). A similar pattern was observed in Urco Miraño, where travelers showed higher diversity (mean π = 0.060; median π = 0) than non-travelers (median π = 0). In contrast, Gamitanacocha showed low diversity in both groups, with slightly lower values among travelers (median π = 0) than non-travelers (median π = 0).

### Genetic differentiation

**Figure 4:**
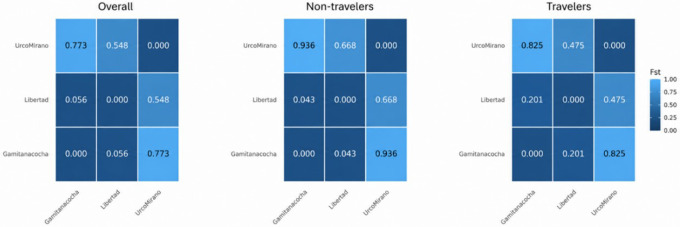
Genetic Differentiation. Heatmaps Fst values for comparing *P. vivax* parasites from Libertad, Gamitanacocha, and Urco Miraño, analysed as overall population, only local infections, and only infections detected in travelers.

For the overall population, the lowest genetic differentiation was observed between Libertad and Gamitanacocha (Fst = 0.05). In contrast, higher differentiation was observed between Urco Miraño and the other communities, with Fst values of 0.548 between Libertad and Urco Miraño and 0.773 between Gamitanacocha and Urco Miraño (Fig. 4).

When the analysis was restricted to non-traveler infections, differentiation between Libertad and Gamitanacocha remained low (Fst = 0.043), indicating continued genetic similarity among resident infections in these two communities. However, differentiation involving Urco Miraño increased, with Fst values of 0.668 between Libertad and Urco Miraño and 0.936 between Gamitanacocha and Urco Miraño.

Among traveler-associated infections, differentiation between Libertad and Gamitanacocha was higher than in the overall and non-traveler datasets (Fst = 0.2), although still lower than comparisons involving Urco Miraño. Traveler-associated infections from Urco Miraño remained highly differentiated from both Libertad (Fst = 0.475) and Gamitanacocha (Fst = 0.825).

Within-community comparisons between travelers and non-travelers showed no statistically significant differentiation in any community (Supplementary Figure S4). The estimated Fst was 0.23 in Gamitanacocha (95% CI: 0.21–0.26; p = 0.41), − 0.07 in Libertad (95% CI: −0.09 to − 0.06; p = 0.96), and 0.06 in Urco Miraño (95% CI: 0.06–0.07; p = 0.91). The negative Fst estimate in Libertad was interpreted as approximately zero, indicating no detectable genetic differentiation between travelers and non-travelers in this community.

### Population structure

**Figure 5:**
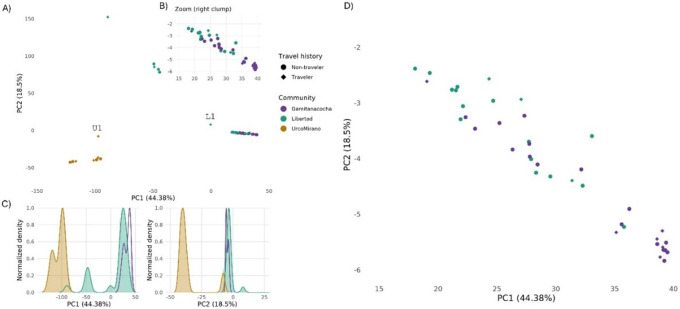
Population structure of *P. vivax* isolates from three communities in the Mazan district based on Principal Component Analysis (PCA). The global distribution of all samples is presented in (A), with a zoomed-in view of the clustering pattern between Libertad and Gamitanacocha in (B). Density distributions along the principal component axes are illustrated in (C), while a higher-resolution view of the overlap between Libertad and Gamitanacocha clusters is provided in (D). U1 and L1 marked on the plots represent traveler-associated isolates with the most genetically distinct profiles compared with the other samples.

PCA revealed clear population structure among *P. vivax* isolates from the three communities (Fig. 5). PC1 explained 44.4% of the total genetic variance and PC2 explained 18.5%, together capturing 62.9% of the overall variation. Urco Miraño samples formed a distinct cluster separated from Gamitanacocha and Libertad, while Libertad and Gamitanacocha were positioned closer together and showed partial overlap (Fig. 4A). In the zoomed view, Gamitanacocha included a compact subgroup, whereas Libertad showed broader dispersion, suggesting greater within-community genetic heterogeneity (Fig. 4B–D). Traveler-associated infections did not form a separate cluster and were generally interspersed within their community-level clusters.

Figure 5. Population structure of *P. vivax* isolates from three communities in the Mazan district based on Principal Component Analysis (PCA). The global distribution of all samples is presented in (A), with a zoomed-in view of the clustering pattern between Libertad and Gamitanacocha in (B). Density distributions along the principal component axes are illustrated in (C), while a higher-resolution view of the overlap between Libertad and Gamitanacocha clusters is provided in (D). U1 and L1 marked on the plots represent traveler-associated isolates with the most genetically distinct profiles compared with the other samples.

**Figure 6:**
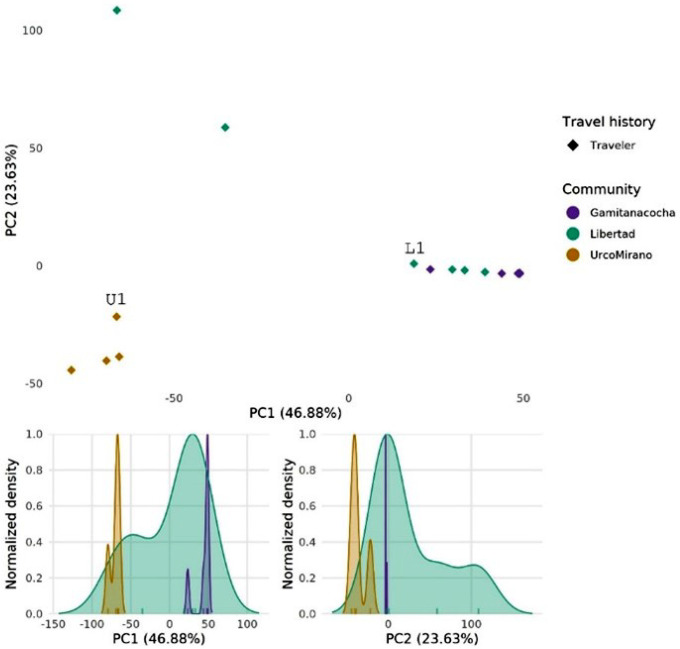
PCA of travel-associated *P. vivax* infection. U1 and L1 marked on the plots represent traveler-associated isolates with the most genetically distinct profiles compared with the other samples.

In the traveler-only PCA subanalysis, traveler-associated infections from Urco Miraño were separated from the other communities, mainly along PC1. In contrast, several infections from Libertad and Gamitanacocha clustered close together with partial overlap, although Libertad showed broader dispersion, including samples separated along PC2 (Fig. 6).

**Figure 7:**
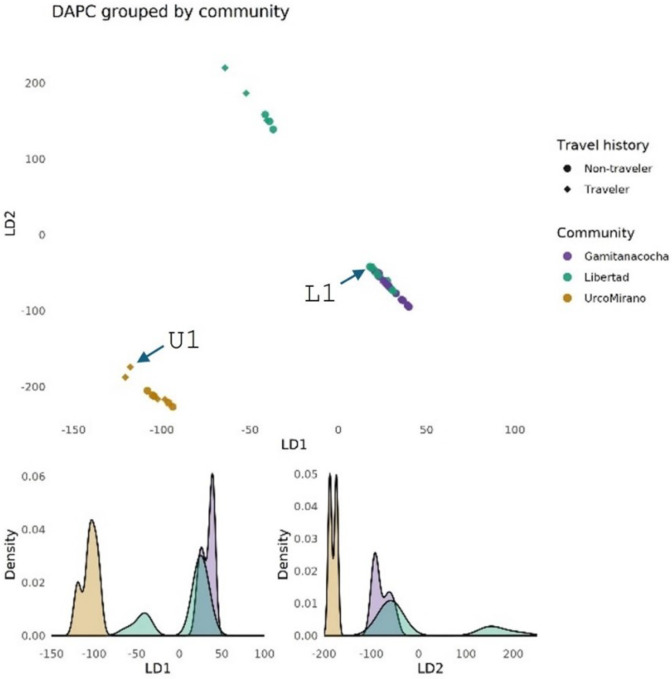
Discriminant analysis of principal components (DAPC) of *P. vivax* isolates from Gamitanacocha, Libertad, and Urco Miraño. U1 and L1 marked on the plots represent traveler-associated isolates with the most genetically distinct profiles compared with the other samples.

DAPC supported the PCA findings, showing clear separation of Urco Miraño from the other two communities, while Gamitanacocha and Libertad were closer but still partially differentiated (Fig. 7). Cross-validation indicated stable assignment performance across a range of retained principal components, and LD loading plots suggested that discrimination was distributed across many SNPs rather than driven by a small number of highly influential loci (Supplementary Figures S5–S6).

**Figure 8:**
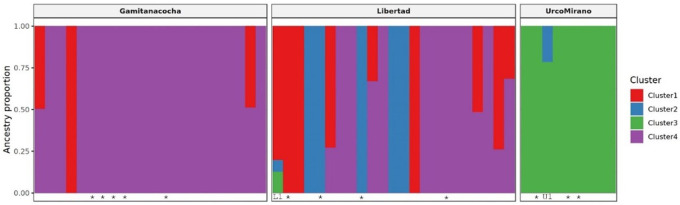
ADMIXTURE analysis (K = 4) of parasite population structure using unpruned SNP dataset. Each vertical bar represents one sample, with colors indicating membership in inferred genetic clusters. Asterisks (*) denote traveler-associated infections. U1 and L1 represent traveler-associated isolates with the most genetically distinct profiles compared with the other samples

ADMIXTURE analysis provided complementary evidence for population structure. Cross-validation identified K = 4 as the optimal number of ancestral populations (Supplementary Figure S7). In the unpruned dataset, Urco Miraño isolates were largely assigned to a single ancestry component, indicating a relatively homogeneous parasite population, whereas Gamitanacocha was also mainly dominated by one different ancestry component with limited admixture. In contrast, Libertad showed greater inter-individual heterogeneity and mixed ancestry patterns (Fig. 8). Notably, one traveler-associated isolates with the most genetically distinct profiles compared with the other samples (L1) showed ancestry contributions from three components, including the component mainly observed in Urco Miraño. In Urco Miraño, only one traveler-associated isolated marked as U1 showed evidence of two ancestry components. The overall ancestry patterns remained consistent after LD pruning at r^2^ = 0.1 and r^2^ = 0.2, supporting the robustness of the observed population structure (Supplementary Figures S8–S9).

### Minimum spanning tree and hierarchical clustering

In the MST, Urco Miraño isolates formed a compact cluster with short connecting edges, indicating high genetic similarity among isolates from this community (Supplementary Figure S10). Libertad showed a more dispersed network structure, with multiple subclusters and longer connecting paths. Gamitanacocha occupied a more central position in the network, with several traveler-associated infections located near central nodes. Overall, traveler-associated infections did not form a separate group and were mostly embedded within community-level clusters, although a small number appeared on peripheral branches.

Hierarchical clustering showed a similar pattern. Urco Miraño formed a distinct and well-defined cluster, separated from the other communities at relatively larger genetic distances. Libertad and Gamitanacocha samples were more interspersed across the dendrogram, forming a broader and more heterogeneous cluster (Supplementary Figure S11). One traveler-associated infection from Libertad formed an isolated branch, suggesting a genetically distinct profile compared with the other samples. Traveler-associated infections were distributed across the dendrogram rather than forming an independent cluster, and many were embedded within clusters dominated by local infections.

### Parasite connectivity

**Figure 9:**
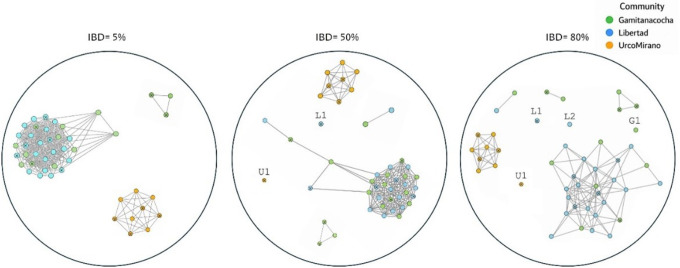
Identity-by-descent (IBD) network of *P. vivax* infections across the three study communities. Each node represents an individual infection, and edges indicate pairs of parasites sharing different levels of identity-by-descent (5%, 50%, and 80%). Crosses (x) denote traveler-associated infections. U1 and L1 represent traveler-associated isolates. L2 and G1 represent non-traveler isolates. The letters indicate the community of origin: L = Libertad, U = Urco Miraño, and G = Gamitanacocha.

At the 5% IBD threshold, which captures background relatedness and older shared ancestry, the network was highly connected, with numerous edges linking samples across different communities. At this threshold, Urco Miraño formed a distinct cluster composed exclusively of isolates from this community, without clustering with samples from Libertad or Gamitanacocha. At 20% IBD, L1 (traveler-associated from Libertad) became a singleton, originated from Cluster 1 (the largest cluster) at 5% IBD. When the threshold was increased to 50%, representing sibling-like relatedness between parasite genomes, the overall number of edges decreased, and the network became more fragmented. Several discrete clusters emerged, and cross-linking between groups was reduced (Fig. 9). At this threshold, a second singleton appeared: U1 (traveler-associated from Urco Miraño), originated from Cluster 2 (the second-largest cluster) at 5% IBD.

At the 80% threshold, representing high genetic relatedness and recent shared ancestry but below the level usually considered near-clonal, the network was markedly sparse and consisted primarily of small clusters, often pairs or tightly connected groups of highly related parasites. Most broader connections observed at lower thresholds were no longer present. At this threshold, two additional non-travelers samples (G1 from Gamitanacocha and L2 from Libertad) emerged as singletons.

**Figure 10:**
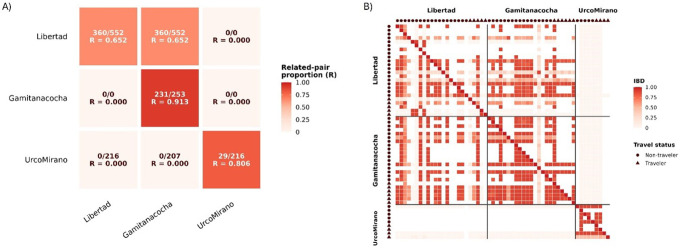
Community- and sample-level patterns of pairwise IBD relatedness among *P. vivax* infections. (A) the proportion of related pairs within and between communities using an IBD threshold of ≥0.50. (B) Sample-level pairwise IBD heatmap, with samples ordered by community and travel status. Circles indicate non-travelers and triangles indicate travelers.

At the community level (Fig. 10), the highest proportion of related pairs was observed within Gamitanacocha (231/253; R = 0.913), followed by within Urco Miraño (29/36; R = 0.806) and within Libertad (180/276; R = 0.652). A similar related-pair proportion was observed between Libertad and Gamitanacocha (360/552; R = 0.652), whereas no related pairs were detected between Urco Miraño and Libertad or between Urco Miraño and Gamitanacocha (Fig. 9A). At the sample level, the pairwise IBD heatmap showed strong relatedness within community blocks and between Libertad and Gamitanacocha, while Urco Miraño showed limited relatedness with the other two communities (Fig. 9B). Travelers were distributed within their respective community blocks and did not form a separate traveler-specific cluster.

## Discussion

In this study, we used WGS data to investigate whether *P. vivax* infections in individuals reporting recent travel outside their community of residence, but within the study region, were genetically differentiated from infections in non-travelers across three communities in the Peruvian Amazon. Across complementary population genetic analysis approaches, we observed different parasite diversity and population structure at community-level and between the local travelers and non-travelers. Urco Miraño was consistently the most genetically distinct and relatively homogeneous population, whereas Libertad showed the highest diversity and strongest evidence of admixture. Gamitanacocha showed lower diversity but remained genetically connected to Libertad, consistent with movement along the shared river system.

Similar fine-scale parasite structure has been reported in other genomic studies, where WGS resolved local differentiation while also revealing connectivity between transmission foci [10, 11]. For *P. vivax*, previous population genomic studies have shown that South American parasite populations often carry signatures of historical bottlenecks, clonal expansion, and relatedness, which can generate local structure while reducing the resolution for assigning infection origin at small spatial [15, 33].

The main question of this study was whether infections in individuals with reported travel could be distinguished from infections in non-travelers. This distinction is important because identifying whether infections are likely linked to local transmission or gene flow between communities can help guide targeted surveillance and malaria elimination strategies [34]. However, distinguishing traveler-associated from locally acquired infections can be challenging in malaria-endemic settings with high human mobility, resulting in high background relatedness, recent shared ancestry, or frequent gene flow. These difficulties have been reported in previous malaria genomic studies, where imported and local infections were difficult to separate without dense sampling and detailed epidemiological data [3, 9]. Consistent with this, traveler-associated infections in our study did not form a single genetically distinct group. Instead, most clustered with parasites from their community of residence, suggesting that reported travel did not necessarily indicate importation and that many infections may have been acquired locally or within the same connected transmission network.

However, not all traveler-associated infections followed this pattern. Two infections, L1 from Libertad and U1 from Urco Miraño, appeared genetically distinct in high-resolution relatedness and network-based analyses. These infections may represent parasite lineages from unsampled locations or less connected transmission networks. This interpretation is consistent with previous work showing that IBD and other haplotype-based analyses can identify recent relatedness and parasite connectivity that may not be fully captured by allele-frequency approaches alone [9, 10]. Nevertheless, IBD should not be interpreted as direct evidence of importation without epidemiological context, because clonal expansion, low recombination, and recent shared ancestry can also generate extensive haplotype sharing in malaria parasite populations [3, 33].

These findings show that the ability of WGS to distinguish traveler-associated from local infections depends strongly on the genetic and epidemiological context. In settings with low parasite diversity, frequent gene flow, and closely related circulating lineages, parasites from different communities may remain genetically similar, reducing the ability to assign infection origin from genomic data alone [15, 33]. This does not indicate that WGS is generally unable to detect imported or mobility-associated infections; rather, it highlights that interpretation requires sufficient regional sampling, detectable population structure, and robust epidemiological data [11, 35]. This challenge is particularly relevant for *P. vivax*, because infections detected after travel may also represent relapses from dormant hypnozoites acquired earlier, including infections originally acquired in the community of residence [16, 17]. Therefore, some travel-associated infections may not reflect recent parasite movement, making interpretation more complex than for *P. falciparum*.

Other analytical factors may also affect interpretation. WGS provides genome-wide genetic variation that can be used to assess parasite relatedness and population differentiation, but the ability to assign infections to specific geographic sources depends on the level of genetic differentiation/similarity among parasite populations and the availability of representative reference data. Previous studies have shown that shared or closely related haplotypes can limit geographic assignment of malaria infections, particularly when parasite lineages are common across regions or when reference sampling is incomplete [36] [37].

Several limitations should be considered when interpreting the findings of this study. First, relying on self-reported travel data may miss some travelers, which can hide or weaken the genomic signals of imported infections. Another important limitation is the potential confounding effect of *P. vivax* hypnozoites. Some infections detected in individuals with reported travel may not necessarily have been acquired during or because of that travel. Instead, they could represent relapses from dormant liver-stage infections acquired earlier, including infections originally acquired in the community of residence. This makes the interpretation of traveler-associated infections more complex than *for P. falciparum*, which does not form hypnozoites [16, 17]. Third, the predominant frequency of genetically related parasites and historical bottlenecks in South American malaria populations may reduce discriminatory power [15, 38]. Fourth, the cross-sectional design and sampling restricted to only three communities limit our ability to infer broader regional parasite connectivity and to assess how parasite populations change over time within these and other surrounding communities. Lastly, the relatively small number of sequenced infections, particularly among traveler-associated infections and in some communities, may have limited the power to detect subtle genetic differences between groups.

Consequently, future studies should incorporate (i) denser regional sampling beyond the three focal communities, (ii) temporal sampling to detect lineage changes, (iii) integration of more accurate human mobility data (e.g., mobile phone data), and (iv) transmission modeling frameworks integrating genetic data, infection timing, travel history, and geographic connectivity to better distinguish local transmission from mobility-associated parasite introductions. Such integrative approaches, combining WGS with robust epidemiological and mobility data, will improve our ability to distinguish imported from local infections and support the development of more targeted malaria elimination strategies.

To conclude, this study confirms the potential of WGS to characterize *P. vivax* population structure across geographically close Amazonian communities. The detection of community-level genetic structure suggests that WGS can provide valuable insight into parasite connectivity at small spatial scales. Most traveler-associated infections clustered with parasite populations in the community of origin, indicating that reported travel does not necessarily imply importation. Instead, many of these infections may have been acquired within the community of residence or from closely connected local transmission networks. However, a small number of traveler-associated infections showed more distinct genetic profiles, suggesting that WGS may help identify potentially mobility-associated parasite lineages when sufficient genetic differentiation is present. Given the limited sample size, particularly in Urco Miraño, these findings should be interpreted cautiously. Similar analytical frameworks, combined with denser sampling and detailed epidemiological data, could be applied in other endemic regions to characterize the genetic profiles of traveler-associated infections and to assess their potential role in introducing and circulating new parasite lineages in settings approaching malaria elimination.

## Supplementary Material

Supplementary Files

This is a list of supplementary files associated with this preprint. Click to download.

- SupplementaryInformation.pdf

## Data Availability

The datasets generated and/or analysed during the current study are not publicly available due to ethical and privacy restrictions related to human participant data. Data supporting the main findings are included in this article and its Supplementary Information files. Additional data may be made available from the corresponding author on reasonable request and subject to approval by the relevant institutional ethics committees.
